# Longitudinal development of the gut microbiota in healthy and diarrheic piglets induced by age‐related dietary changes

**DOI:** 10.1002/mbo3.923

**Published:** 2019-09-09

**Authors:** Qiaoli Yang, Xiaoyu Huang, Pengfei Wang, Zunqiang Yan, Wenyang Sun, Shengguo Zhao, Shuangbao Gun

**Affiliations:** ^1^ College of Animal Science and Technology Gansu Agricultural University Lanzhou China; ^2^ Gansu Research Center for Swine Production Engineering and Technology Lanzhou China

**Keywords:** carbohydrate metabolism, diarrhea, diet, gut microbiota, piglets, weaning

## Abstract

Diarrhea is one of the most common enteric diseases in young piglets. Diverse factors such as an unstable gut microenvironment, immature intestinal immune system, early supplementary feeding, and weaning often induce dysfunction of gut microbiota, thus leading to a continuing high incidence of diarrhea in piglets. However, few studies have characterized the gut microbiota of diarrheic piglets following changes in diet and during the development of intestinal physiology. In this study, we used 16S rRNA gene sequencing to analyze the dynamic establishment of fecal microbiota in six healthy piglets in response to age‐related changes in the diet: sow‐reared, early supplementary creep‐feeding (sow‐reared + starter diet), and weaning (solid nursery diet). We compared the gut microbiota of these six healthy piglets with those of diarrheic piglets during each of the three dietary stages (*n* = 10 sow‐reared, *n* = 10 early supplementary creep‐feeding, and *n* = 5 weaning). We found that weaning (solid nursery feeding) was the primary factor leading to dynamic colonization by microbiota in healthy piglets, and diarrhea primarily affected the microbial communities of piglets before weaning. Healthy piglets showed a continuous decrease in *Lactobacillus* and *Escherichia*, as well as a gradual increase in *Prevotella* with the transition to solid food. An altered relationship between *Prevotella* and *Escherichia* may be the main cause of diarrhea in preweaned piglets, whereas reduced numbers of *Bacteroides*, *Ruminococcus*, *Bulleidia*, and *Treponema* that are responsible for the digestion and utilization of solid feeds may be related to the onset of postweaning piglet diarrhea. The Phylogenetic Investigation of Communities by Reconstruction of Unobserved States (PICRUSt) functional analysis indicated that a reduction in genes involved in carbohydrate metabolism induced by intestinal dysbacteriosis in diarrheic piglets was one of the major causes of diarrhea at the three dietary stages. These findings provide insights into developing an intervention strategy for better management of diarrhea in piglets.

## Graphical abstract

We characterized changing microbial populations in neonatal piglets from suckling the sow, to sow‐suckled plus supplemental creep feeding, and through weaning of piglets to a solid nursery diet in healthy piglets and piglets with diarrhea. Post‐weaning feed is a primary factor influencing the dynamic colonization by microflora in healthy piglets, an obvious continuous decrease in *Lactobacillus* and *Escherichia*, as well as a gradual increase in *Prevotella* in healthy piglets with the transition to solid food. An altered relationship between *Prevotella* and *Escherichia* may be the main cause of diarrhea in pre‐weaning piglets, while reduced numbers of *Bacteroides*, *Ruminococcus*, *Bulleidia*, and *Treponema* may be related to the onset of post‐weaning piglet diarrhea. These findings provide insights for developing an intervention strategy for better management of diarrhea in piglets.

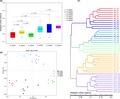

## 1 INTRODUCTION

The gastrointestinal microbiota of mammals play numerous roles in maintaining host health, such as regulating intestinal nutrient metabolism, synthesizing vitamins, promoting the development and maturation of the gut‐associated immune system, and protecting against pathogenic bacteria (Buffie & Pamer, 2013; Kabat, Srinivasan, & Maloy, 2014; Richards, Gong, & Lange, 2005). Diarrhea and gut microbial dysbiosis show reciprocal causation (Zoetendal, Akkermans, Vliet, Visser, & Vos, 2000). In the diarrheic condition, some normal indigenous microflora are discharged and invasive pathogens are abnormally increased, which preferentially lead to a disproportionate amount of beneficial microbes in the gastrointestinal tract; conversely, harmful substances produced by abnormal flora cause abnormalities in the intestinal function and immune response, which in turn cause the development of diarrhea and enteritis (Ward et al., 2016).

Piglet diarrhea is the most common enteric disease in swine, which leads to low growth rates and high mortality rates in neonatal and young piglets. Morris, Davies, and Lawton (2002) reported that almost half (49%) of piglet deaths are caused by diarrhea, which has a detrimental economic impact on the swine industry. To improve the utilization efficiency of sows on modern, intensive pig farms, piglets are usually given supplementary feeding at 1–2 weeks of age and weaned (with a solid nursery diet) at 3 or 4 weeks of age. However, the microenvironment of the gastrointestinal tract is particularly vulnerable to disruptions during the dynamic development of the intestinal mucosal structure and function in young piglets (Isaacson & Kim, 2012). Furthermore, the immune system of piglets undergoes significant postnatal development and has been suggested as a factor influencing the structure of intestinal community (Bailey et al., 2001). These diverse factors often induce the dysfunction of intestinal microflora, thus resulting in the high incidence of diarrhea in piglets.

Several investigations on intestinal microbiota have been focused on dynamic colonization at different ages or following dietary changes in healthy piglets, particularly at weaning (Frese, Parker, Calvert, & Mills, 2015; Slifierz, Friendship, & Weese, 2015). These studies provide evidence that the switch from sows' milk to solid diets critically influences microbial colonization in the immature gastrointestinal tract. Furthermore, several lines of evidence suggest that both specific bacterial species and microbial communities exert either pathogenic effects that facilitate diarrheal disease or probiotic effects that enhance intestinal health (Azcarate‐Peril et al., 2011; Jonach, Boye, Stockmarr, & Jensen, 2014). For instance, the occurrence of neonatal piglet diarrhea is closely related to increases in *Prevotella*, *Sutterella*, *Campylobacter*, and *Fusobacteriaceae*, as well as a decrease in several members of the phylum *Firmicutes* (Hermann‐Bank et al., 2015; Yang et al., 2017). The development of mucohemorrhagic diarrhea in *Brachyspira hyodysenteriae*‐infected piglets was associated with the perturbation of intestinal microbiota, with inoculated diarrheic pigs having lower bacterial numbers and a lower *Bacteroidetes*:*Firmicutes* ratio than uninoculated pigs (Costa, Chaban, Harding, & Hill, 2014). Dietary supplements of *Enterococcus faecalis* LAB31 in weaned piglets may increase the numbers of *Lactobacillus* and consequently reduce the incidence of diarrhea (Hu et al., 2015).

Given these crucial implications, animal scientists have started to perform microbial intervention strategies to improve animal health status, as well as prevent or treat enteric diseases. Zhang et al. (2017) revealed that oral administration of a *Bacillus* mix reprograms the gut microbiota and enhances goblet cell function to ameliorate pig diarrhea caused by enterotoxigenic *Escherichia coli* infection. A recent study indicated that fecal microbiota transplantation (FMT) could reduce susceptibility to epithelial injury and modulate tryptophan metabolism in the microbial community of a piglet model (Geng et al., 2018). Hu, Geng, et al. (2018) and Hu, Ma, et al. (2018) established a standardized model of exogenous FMT in pigs and subsequently found that FMT and oral administration of *Lactobacillus gasseri* LA39 and *Lactobacillus frumenti* strains could modulate the structure of gut microbiota and prevent diarrhea induced by early weaning stress in recipient piglets. In children, microbiota reconstitution has been used to prevent and treat diarrhea caused by *Clostridium difficile* infection (Buffie et al., 2015). Thus, it is conceivable that identification of the gut microbiota in diet‐matched diarrheic piglets could also provide translatable knowledge regarding digestive physiological mechanisms, as well as microbial prevention and therapy for diarrheic disease. However, very few studies have characterized the intestinal microbiota of diarrheic piglets during intestinal development and the associated dietary changes.

In this study, we first characterized the dynamic establishment of individual microbiota in healthy piglets following age‐related dietary changes: sow‐reared (sows' milk), early supplementary creep‐feeding (sows' milk + starter diet), and weaning (solid nursery diet). Second, we examined the differences in the intestinal microbial balance between diarrheic and healthy piglets during these dietary changes. We demonstrated an obvious continuous decrease in *Lactobacillus* and *Escherichia*, as well as a gradual increase in *Prevotella* in healthy piglets with the transition to solid food. An altered relationship between *Prevotella* and *Escherichia* may be the main cause of diarrhea in preweaned piglets, while reduced numbers of *Bacteroides*, *Ruminococcus*, *Bulleidia*, and *Treponema* may be related to the onset of postweaning piglet diarrhea. These findings provide insights for the development of an intervention strategy for better management of diarrhea in piglets.

## 2 MATERIALS AND METHODS

### 2.1 Animals and sample collection

Piglets were the progeny of six third‐parity, healthy Large White sows that were subjected to artificial insemination within 2 days and maintained under identical husbandry practices and epidemic prevention systems on a commercial farm in Gansu province, China, during July 2015. The farm had no previous history of bacterial or viral infections. At birth, the piglets were marked by ear notching for individual identification. They all received identical standards of housing and management, and were exclusively sow‐reared during the first 2 weeks, followed by supplementation with piglet early starter diets at 2 weeks of age. After weaning at 4 weeks of age, piglets were raised on solid nursery feed. The starter diet provided 14.6 MJ/kg digestible energy (DE), 21.0% crude protein, 4.0% crude fiber, 7.0% crude ash, 0.5%–1.2% calcium, 0.55% total phosphorus, 0.3%–1.2% salt, and 1.3% lysine from the 15th to 28th days. For the next 2 weeks after weaning, the diet contained 14.6 MJ/kg DE, 20% crude protein, 4.0% crude fiber, 7.0% crude ash, 0.5%–1.2% calcium, 0.55% total phosphorus, 0.3%–1.2% salt, and 1.45% lysine. The piglets were given ad libitum access to water throughout the experimental period. The general health of each piglet was closely monitored with special attention to fecal consistency and disease history. According to these observations, piglets were defined as diarrheic or healthy and their feces were collected as described previously (Yang et al., 2017). Briefly, diarrheic piglets were those showing signs of diarrhea for at least two consecutive days with thickened and watery feces; healthy piglets never experienced diarrhea or other diseases. Diarrheic samples were collected directly using sterile tools (week 1) or sterile cotton swabs (week 3 and week 5) and immediately frozen in liquid nitrogen.

**Figure 1:**
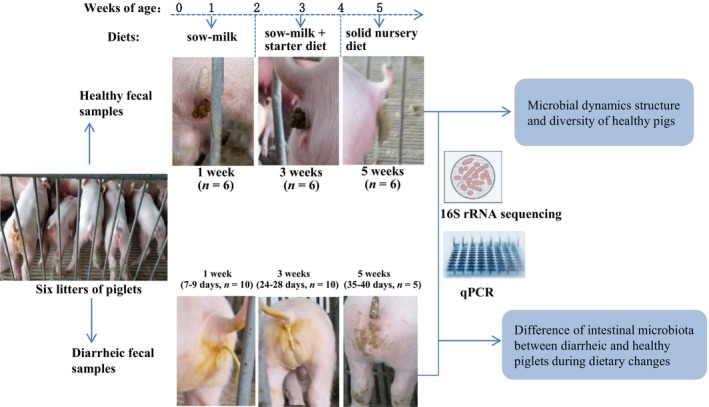
Experimental design of this study

The experimental design of this study is illustrated in Figure 1. To analyze the microbial structure and diversity of healthy piglets at the sow‐reared (sows' milk), early supplementary creep‐feeding (sows' milk + starter diet) and weaning (solid nursery diet) stages, fecal samples from six healthy piglets with similar birth weights and from different litters were collected using sterile cotton swabs at 1 week (H_week1), 3 weeks (H_week3), and 5 weeks (H_week5) of age, respectively. A case–control study was conducted to investigate the fecal microbial profiles of diet‐matched diarrheic and healthy piglets. Diarrheic samples collected at 7–9 days were classified as week 1 (D_week1, *n* = 10), those at 24–28 days were classified as week 3 (D_week3, *n* = 10), and those at 35–40 days were classified into week 5 (D_week5, *n* = 5). The six H_week1 samples and 10 D_week1 samples were also used in our previous study (Yang et al., 2017).

### 2.2 16S rRNA gene sequencing

16S rRNA gene sequencing was used to characterize microbial community diversity and composition in 27 fecal samples of week 3 and week 5 piglets (D_week3 vs. H_week3; D_week5 vs. H_week5). Briefly, bacterial genomic DNA was extracted from fecal samples using a TIANamp stool DNA kit (TIANGEN) according to the manufacturer's instructions. The quality and concentration of samples were estimated using 1% agarose gels and a NanoDrop 2000 spectrophotometer (Thermo Scientific), respectively. The extracted DNA was diluted to 1 ng/μl as a template for PCR using barcoded primers flanking the V4 hypervariable region of bacterial 16S rRNA gene; primer sequences were 515F (5′‐GTGCCAGCMGCCGCGGTAA‐3′) and 806R (5′‐GGACTACHVGGGTWTCTAAT‐3′). PCR reactions were carried out in 25 μl volumes using Phusion^®^ High‐Fidelity PCR Master Mix (New England Biolabs) under the following thermal cycling conditions: one predenaturation cycle at 98°C for 1 min; 30 cycles of denaturation at 98°C for 10 s, annealing at 50°C for 30 s and elongation at 72°C for 60 s; and one postelongation cycle at 72°C for 5 min.

PCR products were purified using the Qiagen Gel Extraction kit (Qiagen). Sequencing libraries were generated using a TruSeq^®^DNA PCR‐Free Sample Preparation kit (Illumina) following the manufacturer's recommendations. Sequencing was performed on an Illumina HiSeq 2500 platform for 2 × 250 bp paired‐end reads at Novogene Bioinformatics Technology Co., Ltd. Paired‐end reads were merged using FLASH (Fast Length Adjustment of SHort reads, Version 1.2.11: http://ccb.jhu.edu/software/FLASH/), and quality filtering of raw reads was performed using the Quantitative Insights into Microbial Ecology (QIIME) pipeline (Caporaso et al., 2010). The acquired sequences were chimera filtered using the UCHIME algorithm (Haas et al., 2011) by aligning them to the reference database (Gold database, http://drive5.com/uchime/uchime_download.html).

### 2.3 Bioinformatics and statistical analyses

High‐quality sequences were assigned to distinct operational taxonomic units (OTUs) using the UPARSE pipeline (v7.0.1001) with a 97% similarity threshold (Edgar, 2013). Representative sequences of each OTU were then taxonomically classified at different levels (phylum, class, order, family, genus, and species) by comparing them to sequences in the GreenGene database (Desantis et al., 2006) using the RDP 3 classifier algorithm (v2.2). The OTU abundance was normalized using a standard with sequence number corresponding to the sample with the least number of sequences.

Alpha diversity measurements (i.e., observed species, Shannon index, Chao1, and ACE) and beta diversity were calculated using QIIME (Caporaso et al., 2010). Intergroup differences in alpha and beta diversity of bacterial communities among treatments (diarrheic vs. healthy) and weeks (1, 3, and 5) were analyzed by a nonparametric test: the Mann–Whitney *U* test was chosen for analysis of two groups, while the Wilcoxon rank‐sum test was chosen for analysis among more than two groups. Dissimilarity matrices of intragroup and intergroup beta distances were visualized using principal coordinate analysis (PCoA) and the unweighted pair‐group method with arithmetic means (UPGMA) analysis. A one‐way analysis of similarity (ANOSIM) test was performed to determine significant differences in bacterial communities among groups using the R software (v3.2.2) (Clarke & Gorley, 2006).

Linear discriminant analysis (LDA) coupled with effect size (LEfSe) was performed to identify the core bacterial taxa differentially represented among healthy piglet groups at the species or higher taxonomic levels. A size‐effect threshold of 4.0 for the logarithmic LDA score was used for discriminative functional biomarkers. Differentially abundant bacterial taxa between diarrheic and healthy piglets at each age (week) stage were detected using the Kruskal–Wallis test, and only taxa with a mean relative abundance >0.1% in at least one group were considered. Significance was considered at *p* < .05.

Functional gene content of the fecal microbiota was predicted using Phylogenetic Investigation of Communities by Reconstruction of Unobserved States (PICRUSt) (Langille et al., 2013) based on taxonomy obtained from the Greengenes (v.13.5) database (DeSantis et al., 2006). Predicted genes were normalized by the 16S rDNA copy number, and their metagenomic contributions were then hierarchically clustered and categorized using the Kyoto Encyclopedia of Genes and Genomes (KEGG) database (Kanehisa et al., 2014).

### 2.4 Quantitative PCR (qPCR)

To assess the results of 16S rRNA gene sequencing, *E. coli*, *Lactobacillus* spp., and *Prevotella* spp., which have been reported to perform crucial functions in maintaining the intestinal health or disease of newborn animals (Bordin et al., 2013; Larsen et al., 2010), were selected for absolute quantification qPCR using previously validated bacterial group‐specific 16S primers (Table A1). The qPCR reactions were conducted in 25 µl volumes containing 12.5 µl 2 × PCR Master Mix, 0.5 µl of each primer, and 1.0 µl template DNA. The reaction conditions included one predenaturation cycle at 94°C for 5 min, 35 cycles of denaturation at 94°C for 30 s, annealing at optimum temperature for 30 s, and elongation at 72°C for 30 s, and one postelongation cycle at 72°C for 10 min. PCR products were purified using a DNA Fragment Quick Recovery Kit (Axygen), ligated into a pMD™19‐T vector (TaKaRa) and transformed into the *E. coli* DH 5a strains according to the manufacturer's instructions. Extraction of plasmid DNA and restriction enzyme digestion from positive clones were used to generate standard recombinant plasmids for each bacterial group. The concentration of standard plasmids was measured using ultraviolet spectrophotometer, and original copy number was calculated.

Standard curves for each target bacteria were generated from the data obtained from qPCR amplification using 10‐fold (i.e., 10^2^–10^7^) serial dilutions of standard recombinant plasmids as templates on an ABI 7500 real‐time PCR system (Applied Biosystems). All amplifications were performed in triplicate. Reactions were conducted in 20 µl volumes containing 10.0 µl SYBR^®^ Premix Ex Taq (2×) Green Master Mix (SYBR green Master Mix, Applied Biosystems), 0.8 µl of each primer, and 1.0 µl template DNA. Amplification curve analyses were performed under with one predenaturation cycle at 94°C for 30 s, 45 cycles of denaturation at 94°C for 10 s, annealing at 60°C for 12 s, and elongation at 72°C for 30 s, and then single‐point signal detection at 72°C. Melt curve analysis was performed immediately after the amplification protocol under the following conditions: 95°C for 15 s, 60°C for 1 min, 95°C for 15 s with acquisition signal detection.

Data obtained from the amplification were transformed to give the number of bacterial log copies/µl feces according to the copy number of recombinant DNA plasmids. The Mann–Whitney *U* test was used for comparisons between two groups.

### 2.5 Spearman's correlation analysis

To capture extreme exclusion relationships among key microbial taxa, Spearman's correlation coefficient (*r*) was analyzed among marker bacterial genera for diarrheic and healthy piglets at week 3 and week 5 stages using the SPSS 18.0 software (SPSS Inc., 2009). Taxa with *r* > .6 or <−.6, and *p* < .05 were considered to have a strong correlation.

## 3 RESULTS

### 3.1 Description of sequencing data

**Figure A1:**
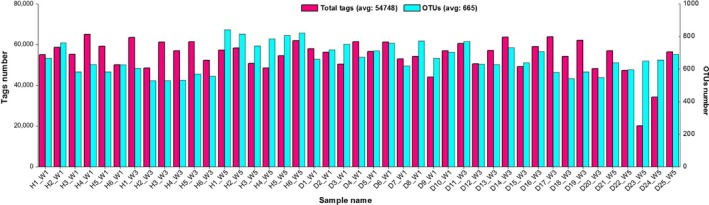
Number of effective tags and OTUs in each fecal sample. The data of six H_week1 samples and ten D_week1 samples were obtained from our previously study (Yang et al., 2017)

**Figure A2:**
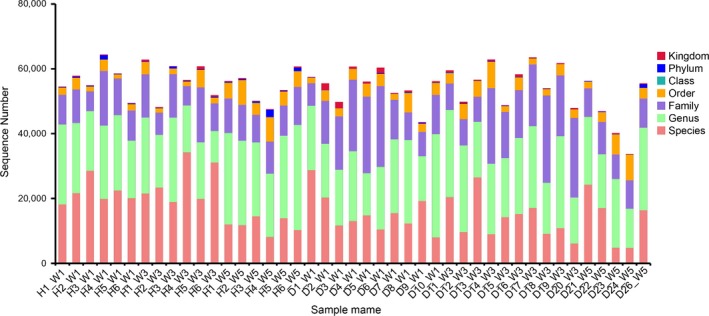
Number of sequences in piglet fecal samples at each taxonomic level. The data of six H_week1 samples and ten D_week1 samples were obtained from our previously study (Yang et al., 2017)

We acquired 2,354,153 high‐quality paired‐end sequences, with an average of 253 bp read length per sample. Based on a 97% species similarity threshold, 1,342 OTUs were identified from all samples, resulting in the classification of 20 phyla, 34 classes, 62 orders, 81 families, and 151 genera. The number of effective tags and OTUs in each fecal sample and the number of sequences in each sample at each taxonomic level are illustrated in Figures A1 and A2. Across all samples, we assigned 99.21% and 68.61% of the total sequences to bacterial phyla and genera, respectively.

### 3.2 Bacterial composition and community diversity associated with age‐related dietary changes in diarrheic and healthy piglets

**Figure 2:**
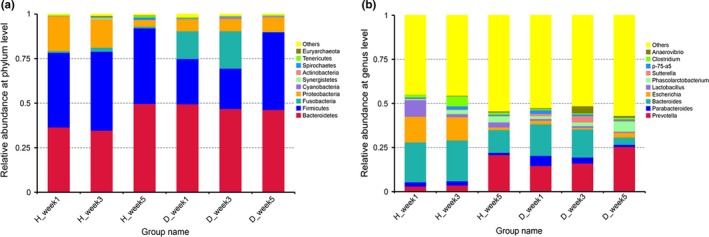
Average relative abundance of the top 10 phyla (a) and the top 10 genera (b) in each piglet group

The relative abundances of the top 10 phyla and top 10 genera present in diarrheic and healthy piglets at different dietary stages are displayed in Figure 2. *Bacteroidetes*, *Firmicutes*, and *Proteobacteria* were the dominant phyla in both diarrheic and healthy piglets; proportions of these reads accounted for more than 95% of reads in healthy piglets at the three dietary stages (H_week1, H_week3, and H_week5), and the weaning diarrheic piglets at week 5 (D_week5). *Bacteroidetes*, *Firmicutes*, and *Fusobacteria* were the dominant microflora in diarrheic piglets before weaning (D_week1 and D_week3) (Figure 2a). Among the annotated genera, 41 had a mean relative abundance of more than 0.1%, accounting for 67.34% of the total bacteria. The dominant phyla and genera in 3‐week‐old piglets were the same as those in 1‐week‐old piglets. *Bacteroides* and *Escherichia* were the most prevalent genera present in healthy piglets at week 3 (H_week3), while *Prevotella* was most prevalent in weaning healthy piglets (H_week5) and diarrheic piglets at week 3 and week 5 (D_week3 and D_week5) (Figure 2b).

The alpha diversity of the fecal microbiota varied considerably following dietary changes in healthy pigs. The richness index (observed species, Chao1 and ACE) of healthy piglets subjected to supplementary creep‐feeding (H_week3) was significantly lower than that of either healthy piglets that were sow‐reared (H_week1) or those subjected to full solid feeding (H_week5) (*p < *.01, Table 1), indicating that sudden dietary changes may perturb the balance in the bacterial community diversity. When compared with healthy piglets that were sow‐reared (H_week1) and supplementary creep‐fed (H_week3), Chao1 and ACE of diarrheic piglets that were sow‐reared (D_week1) and supplementary creep‐fed (D_week3) were both increased (*p* < .05), while the observed species, Chao1, and ACE of diarrheic piglets after weaning (D_week5) were significantly decreased compared with those of healthy piglets (H_week5) (*p* < .01, Table 2).

**Table 1:** Comparison of fecal bacterial α diversity among healthy piglet groups at three age‐related dietary stages

Alpha diversity index	Mean ± *SEM*			Kruskal–Wallis test (*p*‐value)	Mann–Whitney *U* test (*p*‐value)		
	H_week1	H_week3	H_week5		H_week1 versus H_week3	H_week1 versus H_week5	H_week3 versus H_week5
Observed species	499.667 ± 25.264	423.667 ± 11.170	644.667 ± 13.073	0.0009	0.0129	0.0081	0.0050
Shannon	5.854 ± 0.194	5.641 ± 0.174	6.919 ± 0.077	0.0031	0.5887	0.0022	0.0022
Chao1	595.009 ± 23.543	520.983 ± 17.225	777.185 ± 26.145	0.0013	0.0411	0.0022	0.0022
ACE	602.102 ± 24.649	520.659 ± 11.531	783.788 ± 21.174	0.0008	0.0087	0.0022	0.0022

**Table 2:** Comparison of fecal bacterial α diversity between diarrheic and healthy piglet groups at three age‐related dietary stages

Alpha diversity index	Mean ± *SEM*		Mann–Whitney *U* test (*p*‐value)	Mean ± *SEM*		Mann–Whitney *U* test (*p*‐value)	Mean ± *SEM*		Mann–Whitney *U* test (*p*‐value)
	H_week1	D_week1		H_week3	D_week3		H_week5	D_week5	
Observed species	499.667 ± 25.264	544.700 ± 14.403	0.1179	423.667 ± 11.170	484.700 ± 20.365	0.0824	644.667 ± 13.073	546.400 ± 19.057	0.0080
Shannon	5.854 ± 0.194	6.024 ± 0.160	0.6354	5.641 ± 0.174	5.635 ± 0.251	0.8749	6.919 ± 0.077	6.377 ± 0.196	0.0519
Chao1	595.009 ± 23.543	682.561 ± 18.776	0.0160	520.983 ± 17.225	611.110 ± 21.807	0.0075	777.185 ± 26.145	641.164 ± 23.778	0.0087
ACE	602.102 ± 24.649	680.636 ± 16.073	0.0420	520.659 ± 11.531	610.335 ± 21.146	0.0075	783.788 ± 21.174	644.361 ± 2.176	0.0043

**Figure 3:**
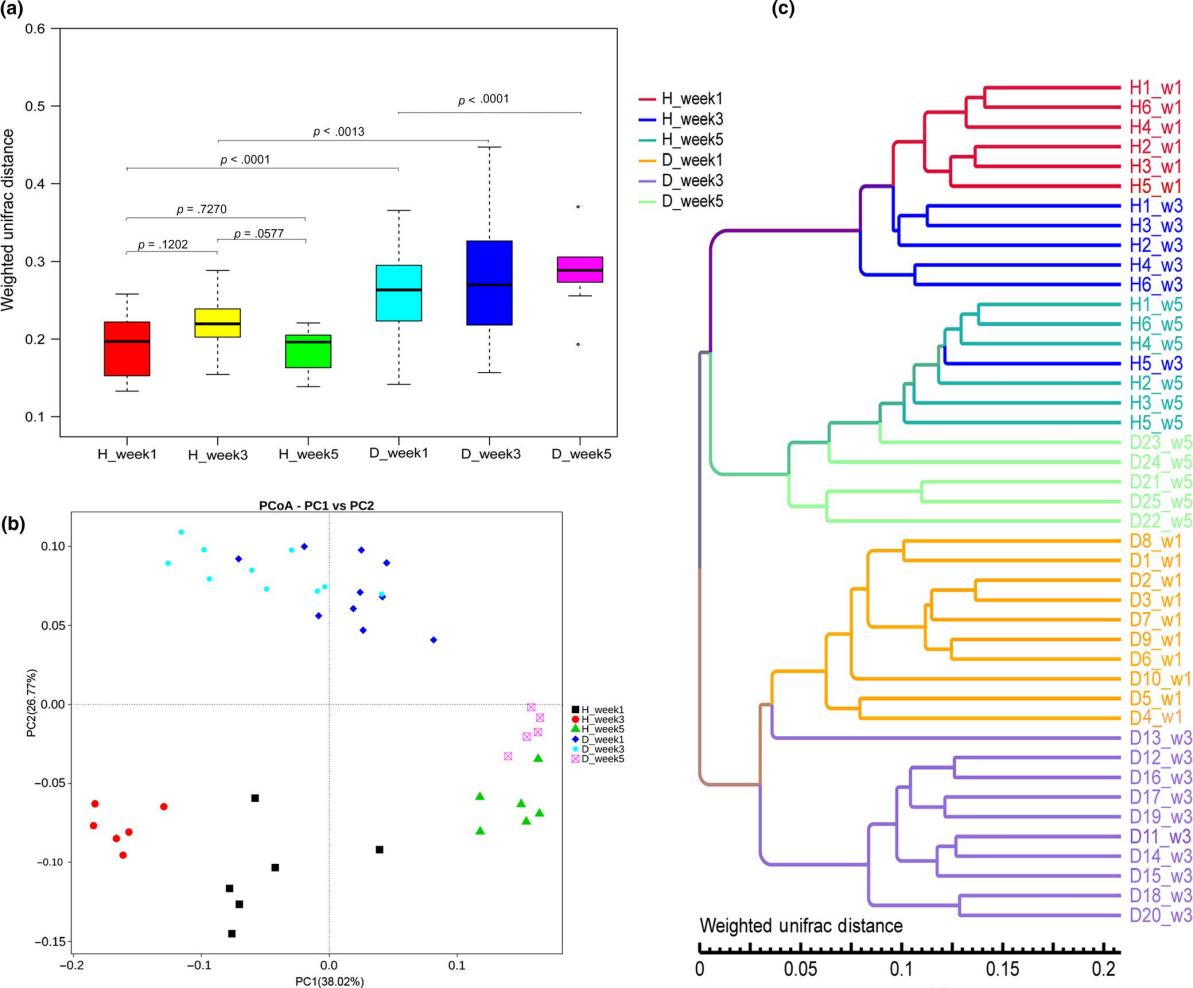
Comparison of fecal microbial community structure of diarrheic and healthy piglets among groups. (a) Boxplot of fecal bacterial β‐diversity (Wilcoxon rank‐sum test, *p* < .01). (b) PCoA analysis of piglet fecal samples based on weighted UniFrac distances (ANOSIM *R*‐value > .5, *p* < .01). (c) Similarity cluster analysis of piglet fecal samples using UPGMA

We found no significant difference in beta diversity of the OTU community structure in healthy piglets among the different sampling stages (Wilcoxon rank‐sum test, *p* > .05). Beta diversity was lower in healthy piglets than in diarrheic piglets at the different sampling stages (Wilcoxon rank‐sum test, *p* < .01; Figure 3a), indicating that diarrhea causes variance in fecal microbial structure and diversity in individual piglets. The principal coordinate analysis (PCoA)‐based trajectory plot also revealed distinct structures among healthy piglets of different ages as well as diarrheic and healthy piglets of the same age, and the ANOSIM for differences was significant (*R*‐value > .5, *p < *.01; Figure 3b). The structure of the gut microbiota became increasingly similar with age and the transition to solid food; before weaning, diarrheic and healthy piglets had distinct microbiota, while there was little difference between the two groups after weaning. Similarity cluster analysis using the UPGMA showed good agreement with PCoA analysis (Figure 3c), indicating the fecal microflora in healthy piglets is ever changing with progressive change of dietary stage, and diarrhea primarily affects the microbial communities in piglets before weaning.

### 3.3 Bacterial biomarkers in healthy piglets at different age‐related dietary stages

**Figure 4:**
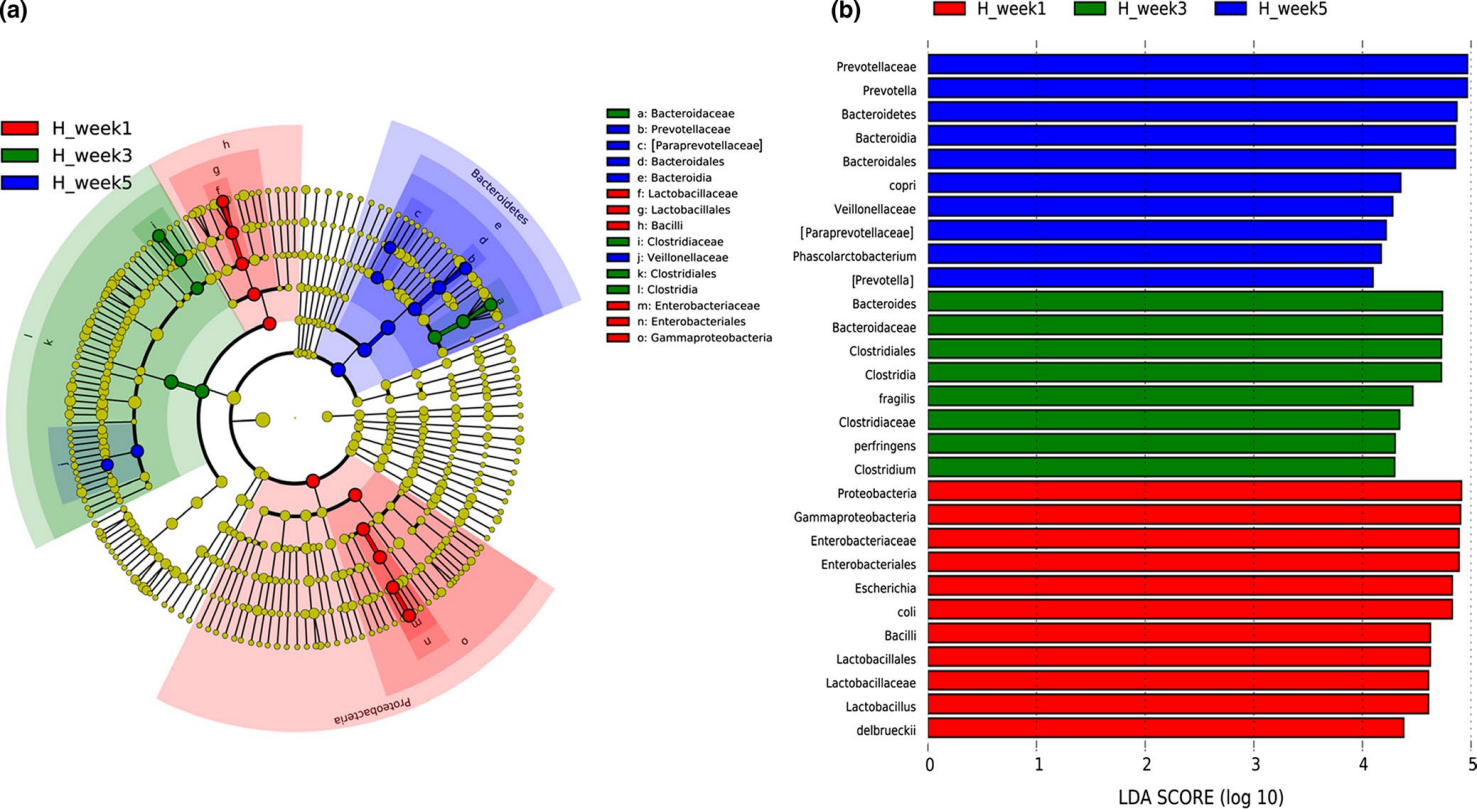
Core biomarkers in healthy piglet groups by LEfSe analysis. (a) The cladogram shows the microbial species exhibiting significant differences in the three groups. Red, green, and blue nodes in the phylogenetic tree represent microbial species that play an important role in the week 1, week 3, and week 5 groups, respectively. (b) Species with significant differences in abundance that have an LDA score > 4.0. The length of the histogram represents the LDA score

We performed LEfSe analysis to reveal the significant ranking of abundant bacterial taxa among H_week1, H_week3, and H_week5 samples. The cladogram in Figure 4a highlights 15 important bacterial taxa, and their significant effects are displayed using LDA scores (Figure 4b). The biomarkers in healthy piglets at 1 week of age were *Bacilli* (h), *Lactobacillales* (g), *Lactobacillaceae* (f), *Lactobacillus*, and *Lactobacillus delbrueckii*, forming one branch; and *Gammaproteobacteria* (o), *Enterobacteriales* (n), *Enterobacteriaceae* (m), *Escherichia*, and *E. coli* forming a second branch.* Clostridium* and *Bacteroides* were biomarkers in healthy piglets at 3 weeks of age. In healthy piglets at 5 weeks of age, the genera *Prevotella/*[*Prevotella*], *Phascolarctobacterium* and the classes *Veillonellaceae* and [*Paraprevotellaceae*] were important biomarkers.

### 3.4 Microbial taxa increased or decreased in diarrheic piglets at different age‐related dietary stages

**Figure 5:**
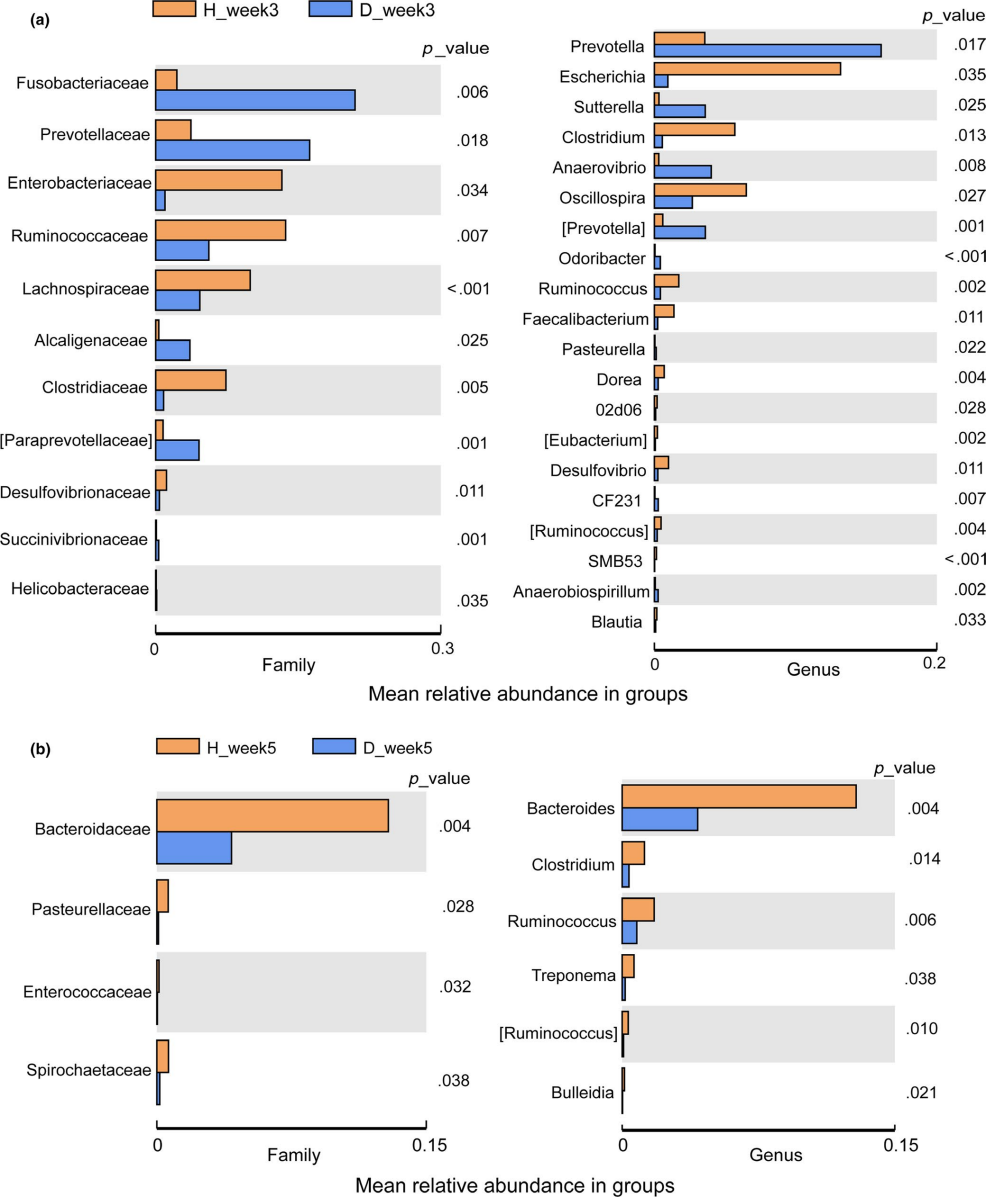
Bacterial taxa showing differences between diarrheic and healthy piglets at 3 weeks (a) and 5 weeks (b) of age. Bacterial taxa with mean relative abundances >0.1% in at least one group are included

We analyzed differences in the major microbial taxa (mean relative abundance > .1% in either of the two groups) between diarrheic and healthy piglets at 3 and 5 weeks of age using the Kruskal–Wallis test (Figure 5).

For piglets at 3 weeks of age, three phyla, 11 families, and 20 genera showed significant differences between healthy and diarrheic piglets (Figure 5a). The relative abundances of the phyla *Firmicutes* (D_week3 vs. H_week3: 0.2269 vs. 0.4435, *p* < .001) and *Euryarchaeota* (D_week3 vs. H_week3: 0.0006 vs. 0.0049, *p* = .04) were significantly decreased in piglets with diarrhea, while the relative abundance of the phylum *Fusobacteria* (D_week3 vs. H_week3: 0.2097 vs. 0.0223, *p* = .006) was significantly elevated in diarrheic piglets compared to healthy piglets. At the genus level, seven genera, *Prevotella*, *Sutterella*, *Anaerovibrio*, *Odoribacter*, *Pasteurella*, *CF231,* and *Anaerobiospirillum*, showed a higher relative abundance in diarrheic piglets than in healthy piglets at 3 weeks of age; *Escherichia*, and nine genera, including *Clostridium*, *Oscillospira*, *Ruminococcus*/[*Ruminococcus*], *Faecalibacterium*, *Dorea*, *02d06*, [*Eubacterium*], *Desulfovibrio*, *SMB53,* and *Blautia*, showed lower relative abundance in 3‐week‐old diarrheic piglets than in healthy piglets at 3 weeks of age (*p* < .05).

For piglets at 5 weeks of age, only the phylum *Spirochaetes* (D_week5 vs. H_week5: 0.0027 vs. 0.0135, *p* = .035); the four families *Bacteroidaceae*, *Enterococcaceae*, *Pasteurellaceae,* and *Spirochaetaceae*; and the five genera *Bacteroides*, *Clostridium*, *Ruminococcus*/[*Ruminococcus*], *Bulleidia*, and *Treponema* showed significant differences between healthy and diarrheic piglets (Figure 5b). The relative abundances of all these bacterial taxa were decreased in diarrheic piglets compared with healthy piglets.

**Figure 6:**
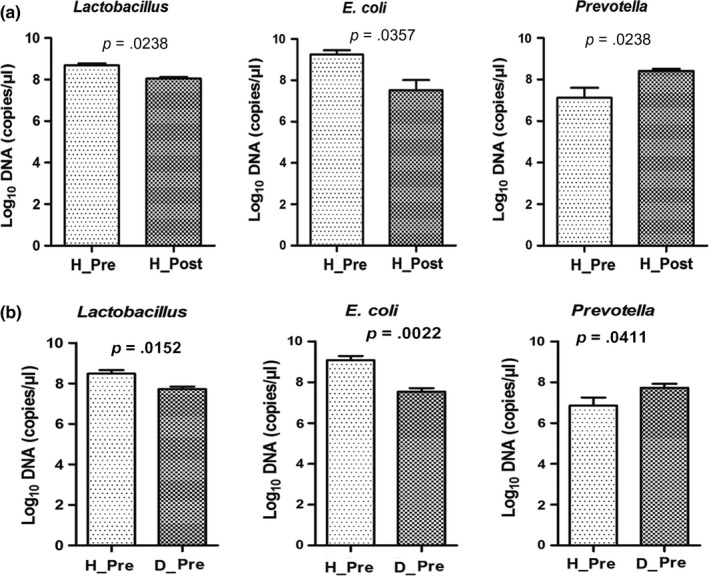
Fecal bacterial concentrations of *Lactobacillus*, *Escherichia coli*, and *Prevotella* quantified by qPCR. (a) Differences in relative abundance between preweaning and postweaning healthy piglets. (b) Differences in relative abundance between healthy and diarrheic piglets before weaning. Significance is considered at *p* < .05

To confirm the results of 16S rRNA gene sequencing, three specific microbial communities (*E. coli*, *Lactobacillus,* and *Prevotella*) were subjected to qPCR (Figure 6). Significant differences in the absolute abundance of *Lactobacillus*, *E. coli,* and *Prevotella* were compared between preweaned and postweaned healthy piglets (Figure 6a). After weaning, the abundance of *Lactobacillus* and *E. coli* was markedly decreased, while the abundance of *Prevotella* was increased in healthy piglets (*p* < .05). The absolute abundance of *Prevotella* in preweaned diarrheic piglets was greater than that in preweaned healthy piglets (*p* < .05), while the abundance of *Lactobacillus* and *E. coli* was significantly lower in preweaned diarrheic piglets than in preweaned healthy piglets (*p* < .05) (Figure 6b). These data were consistent with the results of 16S rRNA gene sequencing, indicating our differential bacterial taxonomy and abundance estimations are highly reliable.

### 3.5 Correlations among bacterial genera in healthy and diarrheic piglets

We performed Spearman's correlation analysis of different bacterial genera of diarrheic and healthy piglets at week 3 and week 5.

For piglets given early supplementary creep‐feed (week 3), a strong negative correlation was observed between *Prevotella* and *Escherichia* in healthy samples (*r* < −.6, *p* < .05), while this correlation was relatively weaker in diarrheic piglets. Diarrhea also caused the correlation between *Lactobacillus* and [*Ruminococcus*] seen in healthy piglets to change from negative to positive (*r* > .6, *p* < .05) (Table 3). This transformation in correlation may be ascribed to the aberration of microbial balance in the diarrheic condition. More understandably, numerous obligate anaerobes with highly similar phylogenic relationships and functions, such as members of the class *Clostridia* (*Ruminococcus*/[*Ruminococcus*], *Blautia*, *Eubacterium*, *Faecalibacterium*, *Oscillospira*, and *Dorea*), showed strong positive correlations in both diarrheic and healthy groups (*r > *.6, *p < *.05). However, only *Clostridium* was positively correlated with [*Ruminococcus*] in postweaned healthy piglets (*r* = .9, *p < *.05), and no significant correlation was noted among other bacterial genera (*p* > .05).

**Table 3:** Spearman's correlation analysis among maker bacterial genera in diarrheic and healthy piglets at 3 weeks of age

Genus		Spearman's correlation coefficient		Genus		Spearman's correlation coefficient	
		Healthy	Diarrhea			Healthy	Diarrhea
*Prevotella*	*Escherichia*	−0.829*	−0.442	*Odoribacter*	*02d06*	0.829*	0.439
*Prevotella*	*Clostridium*	0.543	−0.665*	*Odoribacter*	*CF231*	0.257	0.802**
*Prevotella*	*[Eubacterium]*	−0.257	0.659*	*Odoribacter*	*[Ruminococcus]*	0.714	0.711*
*Prevotella*	*[Ruminococcus]*	−0.086	0.675*	*Odoribacter*	*Blautia*	0.543	0.771**
*Escherichia*	*Clostridium*	−0.371	0.689*	*Ruminococcus*	*Dorea*	0.086	0.948**
*Escherichia*	*Pasteurella*	0.143	0.689*	*Ruminococcus*	*[Eubacterium]*	0.829*	0.756*
*Lactobacillus*	*Sutterella*	0.143	−0.733*	*Ruminococcus*	*CF231*	−0.657	0.766**
*Lactobacillus*	*Anaerovibrio*	0.883*	−0.103	*Sutterella*	*Oscillospira*	−0.543	−0.733*
*Lactobacillus*	*Oscillospira*	−0.829*	0.333	*Sutterella*	*[Prevotella]*	−0.371	−0.709*
*Lactobacillus*	*[Prevotella]*	−0.086	0.770**	*Sutterella*	*Odoribacter*	0.086	−0.939**
*Lactobacillus*	*Odoribacter*	−0.657	0.830**	*Sutterella*	*Ruminococcus*	0.257	−0.818**
*Lactobacillus*	*Faecalibacterium*	−0.754	0.709*	*Sutterella*	*Faecalibacterium*	0.058	−0.709*
*Lactobacillus*	*CF231*	−0.714	0.802**	*Sutterella*	*Dorea*	−0.029	−0.644*
*Lactobacillus*	*[Ruminococcus]*	−0.829*	0.632*	*Sutterella*	*CF231*	−0.143	−0.851**
*Lactobacillus*	*SMB53*	−0.943**	−0.197	*Sutterella*	*[Ruminococcus]*	−0.086	−0.772**
*Lactobacillus*	*Blautia*	−0.886*	0.489	*Sutterella*	*Anaerobiospirillum*	0.265	0.644*
*Oscillospira*	*CF231*	0.486	0.748*	*Sutterella*	*Blautia*	−0.314	−0.838**
*Oscillospira*	*SMB53*	0.943**	0.185	*Clostridium*	*Anaerovibrio*	−0.853*	0.506
*Oscillospira*	*Blautia*	0.886*	0.973**	*Anaerovibrio*	*Faecalibacterium*	−0.851*	−0.321
*[Prevotella]*	*Odoribacter*	−0.257	0.794**	*Anaerovibrio*	*SMB53*	−0.765	−0.745*
*[Prevotella]*	*Ruminococcus*	−0.486	0.648*	*Anaerovibrio*	*Blautia*	−0.853*	−0.367
*Odoribacter*	*Ruminococcus*	0.371	0.794**	*Oscillospira*	*Odoribacter*	0.486	0.636*
*Odoribacter*	*Dorea*	0.886*	0.644*	*Oscillospira*	*Ruminococcus*	−0.086	0.636*
*Oscillospira*	*02d06*	0.771	0.854**	*02d06*	*CF231*	0.143	0.731*
*Ruminococcus*	*[Ruminococcus]*	0.486	0.942**	*02d06*	*[Ruminococcus]*	0.886*	0.465
*Ruminococcus*	*Blautia*	0.257	0.722*	*02d06*	*SMB53*	0.829*	0.328
*Pasteurella*	*Desulfovibrio*	0.829*	−0.25	*02d06*	*Anaerobiospirillum*	−0.912*	−0.41
*Dorea*	*[Eubacterium]*	−0.257	0.765*	*02d16*	*Blautia*	0.771	0.822**
*Dorea*	*[Ruminococcus]*	0.6	0.845**	*[Ruminococcus]*	*[Eubacterium]*	0.486	0.755*
*CF231*	*[Ruminococcus]*	0.2	0.841**	*[Ruminococcus]*	*SMB53*	0.886*	−0.086
*CF232*	*Blautia*	0.371	0.831**	*[Ruminococcus]*	*Anaerobiospirillum*	−0.853*	−0.284
*CF231*	*Faecalibacterium*	0.812*	0.821**	*[Ruminococcus]*	*Blautia*	0.943**	0.672*
*[Ruminococcus]*	*Faecalibacterium*	0.406	0.644*	*SMB53*	*Blautia*	0.943**	0.134

Note

Correlation coefficients ≥ .6 or ≤−.6 either one group with *p* < .05 are showed.

* *p* < .05.

** *p* < .01.

### 3.6 Functional differences of fecal microbiota between diarrheic and healthy piglets

**Figure 7:**
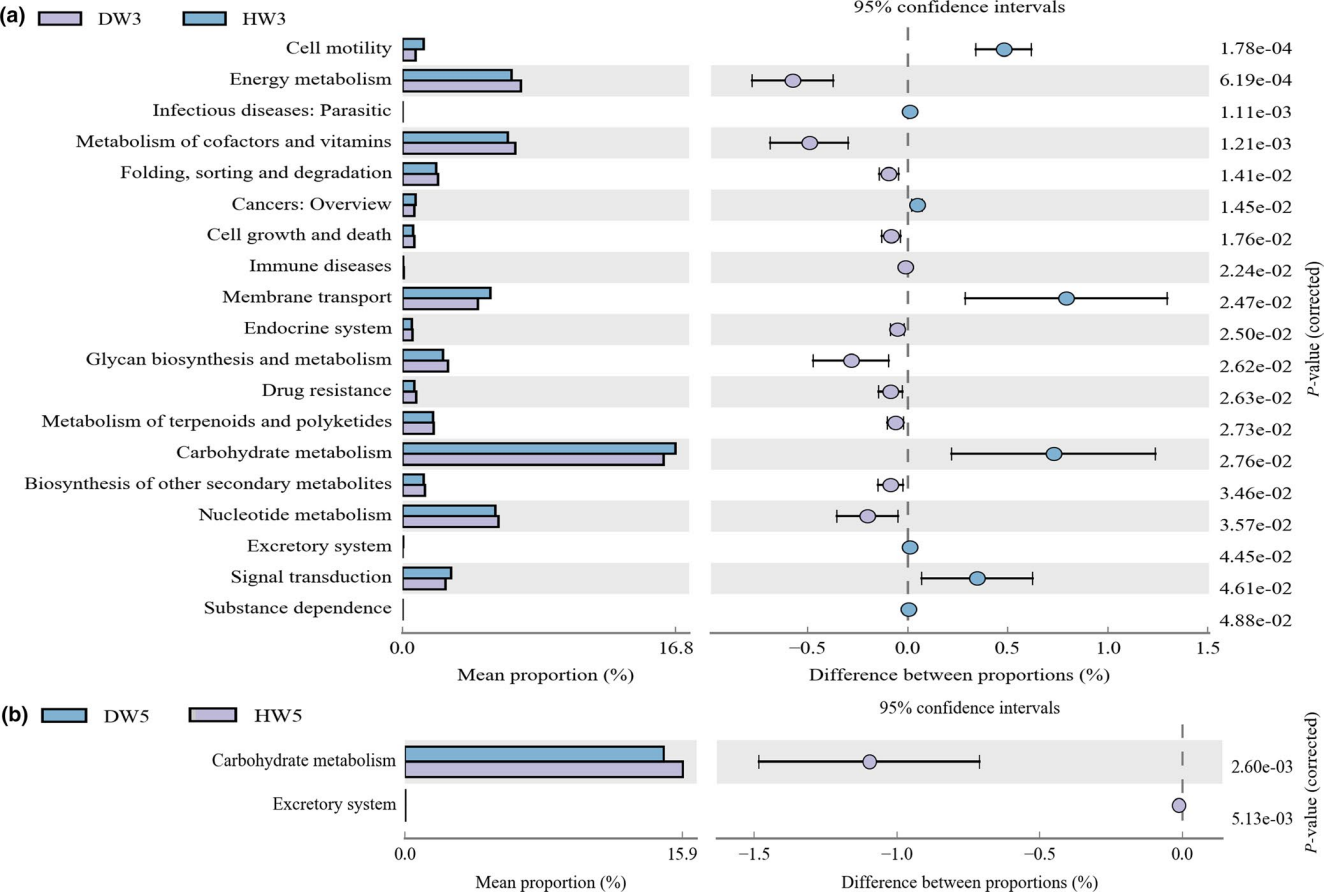
PICRUST analysis of KEGG metabolic pathways at the second level. Graphs show the abundance ratios of different functions between diarrheic and healthy piglets at 3 weeks old (a) and 5 weeks old (b). *p* < .05 represents a significant difference

We compared the functional gene composition of fecal microbiota between diarrheic and healthy piglets using the PICRUSt software. As shown in Figure 7a, notable differences in KEGG pathways between 3‐week‐old diarrheic and healthy piglets included the enrichment of functional genes involved in metabolic pathways, and genetic and environmental information processing (*p* < .05). The metabolic functional genes showing a significantly increased presence in diarrheic piglets were involved in energy metabolism, metabolism of cofactors and vitamins, glycan biosynthesis and metabolism, metabolism of terpenoids and polyketides, and the biosynthesis of other secondary metabolites. In addition, genes associated with nucleotide metabolism were enriched in diarrheic piglets. However, crucial carbohydrate metabolism was reduced in diarrheic piglets. For the genetic and environmental information processing category, functional genes for signal transduction and membrane transport were decreased in diarrheic piglets compared with healthy piglets. At the postweaning stage (week 5), only the functional pathways of carbohydrate metabolism and the excretory system exhibited different gene abundances between diarrheic and healthy piglets (*p* < .05; Figure 7b). These findings suggest that one of the major causes of diarrhea is the reduced uptake and fermentation of available carbohydrates caused by enteric dysbacteriosis in diarrheic piglets.

## 4 DISCUSSION

### 4.1 Microbiota variations in healthy piglets at different age‐related dietary stages

Several studies have shown that the gut microbiome of piglets is rapidly colonized with increasing age and dietary changes (Pajarillo, Chae, Balolong, Bum Kim, & Kang, 2014; Slifierz et al., 2015). In the present study, we analyzed microbial composition and diversity in six healthy piglets at 1, 3, and 5 weeks of age. Alpha diversity was first decreased at 3 weeks of age and then increased at 5 weeks of age, in agreement with the results of Frese et al. (2015), and this is likely linked to the reduction in maternal antibodies absorbed in piglets by 3 weeks old. After weaning, however, with immune system development and the establishment of intestinal microflora of piglets, microbial diversity was increased to accommodate digestion and absorption solid food.

The developing fecal microbiota of piglets in our study was similar to that observed in previous studies, which found *Firmicutes*, *Proteobacteria*, *Bacteroidetes*, *Fusobacteria*, and *Actinobacteria* as the predominant and stable bacterial phyla in piglets (Slifierz et al., 2015). Bacterial diversity and the core microflora of piglets varied with age, with the relative abundance of *Firmicutes* increasing, and the relative abundance of *Proteobacteria*, *Fusobacteria,* and *Actinobacteria* decreasing as piglet age increased (Slifierz et al., 2015). At the genus level, we found that *Lactobacillus*, *Escherichia,* and *Clostridium* were more abundant in preweaned healthy piglets than in postweaned piglets. *Escherichia* and *Lactobacilli* are commonly found in newborn animal intestines (Collado, Cernada, Baüerl, Vento, & Pérez‐Martínez, 2012). *Escherichia coli* is a representative species of the genus *Escherichia*; most *E. coli* strains are harmless and exert a barrier effect against colonization by intestinal pathogenic bacteria (Hudault, Guignot, & Servin, 2001), producing vitamin B and K2 for their hosts (Meganathan, 2001). *Lactobacilli* can protect against enteric pathogens by competing for nutrients and mucosal binding sites, and producing antibacterial substances (i.e., lactic acid, bacteriocin, etc.) (Li et al., 2012; Mann et al., 2014). Liu et al. (2017) reported that *Lactobacillus casei* can improve porcine intestinal immunologic function and consequently reduce the number of cases of piglet diarrhea and death. After weaning, the proportion of *Lactobacillus* in the gut microbiome of piglets declines (Pajarillo, Chae, Balolong, Kim, et al., 2014; Su, Yao, Perez‐Gutierrez, Smidt, & Zhu, 2008). In the present study, *Lactobacillus* and *Escherichia* were the core microflora of piglets at 1 week of age, indicating that they play crucial roles in establishing and maintaining the intestinal flora of piglets after birth.

Several strains representing the genus *Clostridium* are butyrate producers, which convert oligosaccharides (i.e., soybean fibers, fructo‐oligosaccharide, and maltose) to organic acids and alcohols, and thus maintain the balance of intestinal microflora (Louis & Flint, 2009). Additionally, *Clostridium* can modulate systemic immune responses by inducing the expansion of regulatory T_regs_ and the production of inflammatory cytokines, and is associated with colitis resistance (Atarashi et al., 2011). Similar to the finding of Gorham, Williams, Gidley, and Mikkelsen (2016), we found that *Clostridium* was a core bacterial genus in piglets after supplementary creep‐feeding (3 weeks), suggest that an increase of *Clostridium* is conducive for the digestion of cellulose and hemicellulose substrates in piglets. Alternatively, certain dietary constituents provide main energy source or affect gastrointestinal acidity for the growth of gut microbiota, and it is likely a more pH neutral digesta is more conducive for *Clostridium* to thrive in the intestine.

Although Slifierz et al. (2015) found that *Megasphaera* and *Lactobacillus* were the predominant genera in postweaned healthy piglets, there is evidence that *Prevotella* spp. dominate swine fecal microbial communities and gradually increase in numbers with age (Lamendella, Domingo, Ghosh, Martinson, & Oerther, 2011; Pajarillo, Chae, Balolong, Bum Kim, et al., 2014). *Prevotella* is a key microbial member of the gastrointestinal tracts of adult animals; it is crucial for the degradation of starch and plant polysaccharides but also has a strong capacity for mucoprotein catabolism (Ivarsson, Roos, Liu, & Lindberg, 2014; Rho et al., 2005). Frese et al. (2015) revealed that the relative abundance of *Prevotellaceae* increased nearly 50‐fold from an average of 0.3% in suckling piglets to 14.8% in weaned piglets. We found a low relative abundance of *Prevotella* in preweaned healthy piglets and, typically, a much higher abundance in postweaned healthy piglets. This difference is believed to be linked to a diet containing less‐digestible solid feed after weaning, suggesting that the increase in *Prevotella* is likely conducive to digestion and the utilization of feed nutrients in weaned piglets.

### 4.2 Differences in fecal microbiota between diarrheic and healthy piglets

Abundant evidence has revealed an intimate biological interaction between host animals and their gut microbiota. Pop et al. (2014) showed that fecal microbial diversity gradually increases with age and that children with diarrhea have lower microbial diversity than healthy children. In this study, analysis of microbial diversity revealed a higher diversity index in preweaned diarrheic piglets (D_week1 and D_week3) than in preweaned healthy piglets (H_week1 and H_week3). However, the diversity index in postweaned diarrheic piglets (D_week5) was lower than that in postweaned healthy piglets (H_week5). These results suggest that early supplementary creep‐feeding has little effect on microbial diversity and composition before weaning, while the imbalanced microbial diversity observed in diarrheic piglets is correlated with particular changes in their solid feed after weaning.

Earlier findings underscore the importance of microbial composition variation during the development and progression of intestinal diseases (Kamada, Seo, Chen, & Núñez, 2013). In this study, differences were also observed in bacterial species and their relative abundance between diarrheic and healthy piglets at three age‐related dietary stages, especially in those receiving early supplementary creep‐feed (H_week3 vs. D_week3). Furthermore, most of these major differences in bacteria coincided with those identified in our previous study in healthy and diarrheic piglets receiving full sows' milk (H_week1 vs. D_week1) (Yang et al., 2017). Diarrheic piglets showed decreased relative abundance of *Firmicutes*, the major genera being *Ruminococcus*/[*Ruminococcus*], *Anaerovibrio*, and *Clostridium*, which is consistent with previous research on neonatal healthy and diarrheic piglets (Hermann‐Bank et al., 2015). Several members of the phylum *Firmicutes* are believed to produce short‐chain fatty acids (SCFAs), related to energy acquisition and immune regulation (Atarashi et al., 2011). For instance, several members of *Clostridiales* are metabolized to produce butyrate (Devillard, Mcintosh, Duncan, & Wallace, 2007; Louis & Flint, 2009). Reduced abundance of these main *Firmicutes* types in the fecal microbiome of diarrheic piglets may concomitantly affect their digestive physiology and immune functions.

The phylum *Proteobacteria* including members of the genera *Sutterella* and *Campylobacter* is reportedly associated with intestinal inflammatory disease (Minamoto et al., 2014). In dogs with acute diarrhea, the relative abundances of *Sutterella* and *Clostridium perfringens* are decreased while those of *Blautia*, *Faecalibacterium*, and *Turicibacter* are increased when compared with levels in healthy dogs (Suchodolski et al., 2012). These findings concur with our results that *Sutterella* and *Campylobacter* showed increased relative abundance in preweaned diarrheic piglets, suggesting these two genera may be considered potential pathogens in the etiology of piglet diarrhea.

Research has shown that the co‐occurrence of *E. coli* and *Enterococcus* increases the risk of neonatal piglet diarrhea (Hermann‐Bank et al., 2015; Jonach et al., 2014). The predominant bacterial clades containing *Gammaproteobacteria*, *Escherichia,* and *E. coli* were significantly reduced in diarrheic piglets receiving both early supplementary creep‐feed and sows' milk; thus, we believe this may cause the reduced numbers of *Proteobacteria*. Furthermore, *E. coli* have been reported to have unique functions in cell adhesion, oxidative phosphorylation modulation, and protein synthesis (Vlasblom et al., 2015). For instance, the probiotic *E. coli* Nissle 1917 has been demonstrated to limit the expansion of competitors such as adherent‐invasive *E. coli* and the related pathogen *Salmonella enterica* in the inflamed intestine and prevent infectious diarrhea (Sassone‐Corsi et al., 2016). Based on these findings, it is understandable that the *E. coli* identified in the preweaned piglets of the present study is a nonpathogenic microorganism that confers benefits to the host.

Although *Prevotella* has important roles in nutrient digestion and absorption (Ivarsson et al., 2014; Sandberg, Kovatcheva‐Datchary, Björck, Bäckhed, & Nilsson, 2018), its pro‐inflammatory effect of *Prevotella* has been gaining increasing attention as it is correlated with enhanced susceptibility to arthritis (Hofer, 2013). Increases in the abundance of *Prevotella copri* correlate with reduction in the abundance of several beneficial microbes, such as *Bacteroides*, *Clostridium* cluster XIV, *Blautia*, and *Lachnospiraceae*, resulting in reduced resistance to colitis (Atarashi et al., 2011; Scher et al., 2013). Intriguingly, we obtained similar results to Scher et al. (2013) in that *Prevotella* had a greater relative abundance in diarrheic piglets than healthy piglets receiving early supplementary creep‐feed or sows' milk. This appears to be much easier for the colonies of *Prevotella* to become established in conditions of enteric disease than in normal intestinal conditions. Studies have indicated that *Prevotella* can help to reduce the risk of gastrointestinal disorders in children by inhibiting pathogenic *Escherichia* (De et al., 2010), particularly in diarrhea (Kang et al., 2013; Pop et al., 2014).

The disruption of competitive relationships between *Prevotella* and *Escherichia* was found to be associated with neonatal piglet diarrhea (Yang et al., 2017). These observations highlight attenuated or altered bacterial inter‐relationships in individuals with diarrheal disease; thus, synergistic or competitive relationships of intestinal bacteria are closely related to microbial diversity and health status in the intestinal microenvironment. Indeed, our evaluation of correlations among marker genera associated with piglet diarrhea during early supplementary creep‐feeding showed consistent results with the study of neonatal piglets receiving sows' milk (Yang et al., 2017). Therefore, we consider that *Prevotella* confers a high risk for preweaning piglet diarrhea, resulting in decreases in *E. coli* and beneficial *Firmicutes*, which maintain both a balance in the gastrointestinal microecological environment, and the normal function of the mucosal barrier and immunity.

The current consensus is that *Fusobacterium* is a common pathogen of the gastrointestinal mucosa, the high abundance of which is associated with increased expression of inflammatory factors (Nosho et al., 2016). The relative abundance of *Fusobacteriaceae* was higher in animals with neonatal porcine diarrhea or horse colitis than in healthy subjects (Costa et al., 2012; Hermann‐Bank et al., 2015). It is noteworthy that *Fusobacterium* is a small group with low abundance in the intestinal tract of humans and animals, and this is possibly the reason it has not been specifically reported in these studies possibly. Uniformly, we found that the proportion of members of the *Fusobacteriaceae* family in the fecal microbiome of diarrheic piglets was higher than that in both neonatal healthy piglets (Yang et al., 2017) and healthy piglets receiving early supplementary creep‐feed. This suggests that the *Fusobacteriaceae* group may be a valuable diagnostic indicator of preweaning piglet diarrhea.

When compared to postweaned healthy piglets, only the *Spirochaetes* phylum and five genera (*Bacteroides*, *Clostridium*, *Ruminococcus*/[*Ruminococcus*], *Bulleidia*, and *Treponema*) showed lower relative abundance in postweaned diarrheic piglets. These genera are all key participants in nutrient metabolism, including carbohydrate fermentation, and polysaccharide and steroid metabolism, and are crucial for maintaining normal physiological function of the intestine (Flint, Bayer, Rincon, Lamed, & White, 2008; Hess et al., 2011; Louis & Flint, 2009). *Treponema* and *Bulleidia* are low‐abundance microflora in the intestinal tracts of animals and humans. Our results concur with those of a previous study in which the relative abundance of *Treponema* accounted for 3%–4% of the fecal microbiome of 6‐month‐old healthy pigs (Lamendella et al., 2011). Taken together, a large discrepancy was noted in the fecal microflora associated with diarrhea between preweaned (sow‐reared and early supplementary creep‐fed) and postweaned piglets. As the diet of piglets is completely transformed to solid foodstuffs after weaning, a reduction in the numbers of beneficial bacteria reduces the ability of intestinal epithelial cells to digest and absorb proteins and carbohydrates, which in turn leads to permeability disorders and diarrhea.

The PICRUSt analysis of the fecal microbial function between diarrheic and healthy piglets gave similar results to the aforementioned analyses. Diarrhea caused by an intestinal microbiota disorder significantly changed the specific metabolic pathways present in preweaned piglets, which is consistent with the results of another study on diarrheic captive musk deer (Li et al., 2018). Compared with healthy piglets, the most obvious change in diarrheic piglets was the reduced abundance of functional genes involved in carbohydrate metabolism at all three age‐related dietary stages. Many of the bacterial taxa that showed reduced numbers in piglets with diarrhea are associated with the production of SCFAs from carbohydrates, which has also been observed in humans with Crohn's disease (Erickson et al., 2012), suggesting that carbohydrate dysmetabolism may be an important feature of piglet diarrheal disease. It may be as a consequence of increased levels of other important functions like energy metabolism, the metabolism of vitamins, cofactors, or terpenoids and polyketides, or glycan biosynthesis in preweaned diarrheic piglets.

## 5 CONCLUSIONS

Our study identified additional characteristics of the gut microbiota in diarrheic piglets during age‐related dietary changes and the development of intestinal physiology. Postweaning feed is a primary factor influencing the dynamic colonization by microflora in healthy piglets. Obvious changes include a continuous decrease of *Lactobacillus* and *Escherichia*, as well as a gradual increase of *Prevotella* during the transition to a solid diet. Piglets receiving sows' milk or early supplementary creep‐feed had identical major microbial communities that were associated with diarrhea. An altered relationship between *Prevotella* and *Escherichia* may be the main cause of diarrhea. Reduced numbers of *Bacteroides*, *Ruminococcus*, *Bulleidia,* and *Treponema* may be related to the onset of postweaning piglet diarrhea. These findings provide insights for the development of intervention strategies for better management of diarrhea in piglets. However, further studies should be considered that are focused on the reciprocal interactions between gut microbiota and host genetics and/or metabolic substances or the intestinal immune system in response to microbial alterations.

## CONFLICT OF INTERESTS

None declared.

## AUTHOR CONTRIBUTIONS

Conceptualization: Shuangbao Gun. Funding Acquisition: Shuangbao Gun. Investigation: Qiaoli Yang, Xiaoyu Huang, Pengfei Wang, Zunqiang Yan, Wenyang Sun. Writing – Original Draft Preparation: Qiaoli Yang. Writing – Review & Editing: Qiaoli Yang, Shengguo Zhao.

## ETHICS STATEMENT

All animal experimental protocols were conducted according to the guidelines for the care and use of experimental animals established by the Ministry of Science and Technology of China (Approval number 2006‐398). The project was approved by the Institutional Animal Care and Use Committee (IACUC) of Gansu Agricultural University.

## Data Availability

The datasets generated and/or analyzed during the current study are available in the NCBI sequence read archive (SRA) database at https://trace.ncbi.nlm.nih.gov/ under BioProject number of SRP083116 and BioSample number of SRS2068524 and SRS1651658.

## APPENDIX 1

**Table A1:** Specific primer targeting bacteria used for real‐time quantitative PCR

Bacteria	Name	Sequence (5′–3′)	Size (bp)
*Prevotella*	Prev_F	CACCAAGGCGACGATCA	283
	Prev_R	GGATAACGCCYGGACCT	
*Escherichia coli*	E.coli_F	GTTAATACCTTTGCTCATTGA	340
	E.coli_R	ACCAGGGTATCTAATCCTGTT	
*Lactobacillus*	Lab_F362	AGCAGTAGGGAATCTTCCA	341
	Lab_R677	CACCGCTACACATGGAG	
